# Rainfall-Driven Mobilisation of Clinically Relevant *Burkholderia pseudomallei* in a Groundwater-Connected Urban Creek, Northern Australia

**DOI:** 10.3390/pathogens15030276

**Published:** 2026-03-03

**Authors:** Kaitlin Janssen-Groesbeek, Jennifer Elliman, Catherine Rush, Jeffrey Warner

**Affiliations:** Biomedical Sciences and Molecular Biology, College of Medicine and Dentistry, Australian Institute of Tropical Health and Medicine, James Cook University, Townsville, QLD 4811, Australia; kaitlin.burns@my.jcu.edu.au (K.J.-G.); jennifer.elliman@jcu.edu.au (J.E.); catherine.rush@jcu.edu.au (C.R.)

**Keywords:** *Burkholderia pseudomallei*, melioidosis, groundwater, genomic epidemiology, multi-locus sequence type

## Abstract

*Burkholderia pseudomallei* is a saprophytic environmental bacterium and the causative agent of melioidosis, a serious opportunistic infection in tropical regions, including northern Australia. Infection occurs following environmental exposure via percutaneous inoculation, ingestion, or inhalation; however, the environmental reservoirs and transmission pathways responsible for human disease remain poorly defined. Groundwater has been implicated as a potential source of infection, but the factors influencing the persistence and mobility of *B. pseudomallei* in surface waters in North Queensland are not well understood. Water samples were collected from a groundwater-connected seasonal creek in Townsville, North Queensland, over a 12-month period encompassing wet and dry seasons. Samples were cultured on Ashdown agar and confirmed as *B. pseudomallei* by qPCR. Multi-locus sequence typing (MLST) was performed using targeted allele sequencing on the Oxford Nanopore MinION platform. Eighteen of 59 water samples were culture-positive for *B. pseudomallei*. Detection occurred exclusively in turbid, flowing water following ≥30 mm of rainfall and was observed in both wet and dry seasons. MLST of 48 isolates identified 18 sequence types, including 12 novel types. Six sequence types matched previously reported Townsville clinical isolates. These findings indicate that groundwater from a connected urban creek may function as a mobile reservoir for clinically relevant *B. pseudomallei* strains under specific hydrological and climatic conditions, highlighting rainfall-driven processes as key drivers of environmental exposure risk.

## 1. Introduction

*Burkholderia pseudomallei* is a Gram-negative environmental bacterium and the causative agent of melioidosis, a potentially severe opportunistic infection of humans and animals in tropical regions. Infection follows exposure to environmental *B. pseudomallei* via inoculation, ingestion, or inhalation, yet the specific reservoirs and transmission pathways responsible for human disease are often difficult to identify [1,2,3]. In northern Australia, melioidosis remains a major public health concern, with incidence increasing in recent years [4], underscoring the need to better define environmentally driven exposure pathways.

Groundwater is now recognised as a key environmental reservoir and exposure pathway for *B. pseudomallei* in northern Australia [5,6,7,8,9]. Repeated detection of the organism in groundwater discharging to surface watercourses implicates groundwater–surface water interfaces as important points of human and animal contact [5,8,10]. Hydrologic settings that promote discharge, including springs, seepages, and bank outflows, provide plausible exposure routes consistent with observed epidemiological patterns [7]. In North Queensland, the spatial clustering of melioidosis has been linked to geology, soil characteristics, and surface and subsurface water movement, emphasising the role of catchment hydrology in governing mobilisation and availability of *B. pseudomallei* [11,12,13].

International studies reinforce the importance of hydrologically mediated exposure. In Laos, rainfall-driven runoff has been used to characterise *B. pseudomallei* distribution and genetic diversity, demonstrating mobilisation into surface waters during wet conditions [14]. Melioidosis incidence in Northeast Thailand shows strong seasonal variation correlating with monsoon rainfall, where rice paddy water and runoff fields are primary reservoirs for human exposure [15,16]. In Papua New Guinea, lagoons and near-shore aquatic environments have been implicated as exposure settings, highlighting the role of aquatic habitats alongside soils as pathogen reservoirs [17,18]. Across Australia, landscape modification and altered recharge–runoff regimes further influence *B. pseudomallei* persistence and transport, particularly in groundwater-connected systems [9,19,20,21].

Climatic variability strongly modulates these hydrologic connections and melioidosis risk. Heavy rainfall is associated with increased incidence in Darwin, Northern Territory [22], while in Townsville, North Queensland, humidity and rainfall, particularly prolonged rainfall, are important predictors of case occurrence [23]. Queensland surveillance data demonstrate marked interannual variability in notifications, with sharp increases during unusually wet years [24,25]. Climate projections indicate increasing rainfall intensity and flash-flood risk across northern Australia [26], conditions likely to enhance aquifer–stream exchange and expand environmental exposure to *B. pseudomallei*.

Despite growing recognition of groundwater as a reservoir, the temporal dynamics of *B. pseudomallei* mobilisation and persistence within seasonal surface-groundwater systems remain poorly characterised, particularly in urban and peri-urban North Queensland. Seasonal creeks represent dynamic interfaces where groundwater discharge, runoff, and sediment transport converge, yet their role as transient exposure pathways has not been systematically examined.

This study characterises the temporal dynamics of *B. pseudomallei* in a seasonal creek in northern Queensland, focusing on rainfall, water flow, and turbidity, and assesses the clinical relevance of environmental isolates using genomic epidemiology. By integrating longitudinal environmental sampling with sequence-based analysis, this work clarifies how hydrologically driven processes generate short-term exposure risk in groundwater-connected urban waterways.

## 2. Materials and Methods

### 2.1. Longitudinal Study at Selected Location

**Figure 1:**
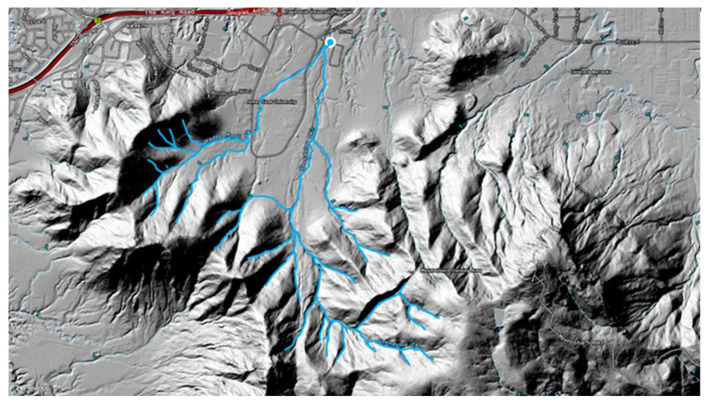
Waterways flowing off Mt Stuart that converge into Goondaloo Creek drain catchment sampling site (◎). Waterways are indicated in blue. Map shows elevation using traditional hillshade. Map created in Queensland Globe [27]. Map copyright © The State of Queensland (Department of Natural Resources and Mines, Manufacturing and Regional and Rural Development) 2025. Map licensing © CNES reproduced under license from Airbus DS, all rights reserved © 21AT © Earth-i, all rights reserved, © Planet Labs PBC, 2025.

**Figure 2:**
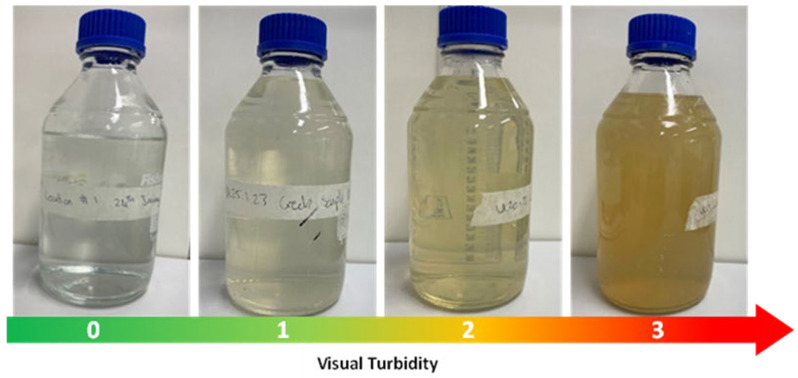
Visual turbidity scale of groundwater collected from Goondaloo Creek.

A total of four sample sites were initially screened for suitability for use in this study. The selection criteria and results of selection are included in Supplementary Materials S1–S8. The inclusion criteria comprised detection of high concentrations of *B. pseudomallei*, proximity of the sampling site to James Cook University, and representation of a broad hydrological catchment, defined as multiple upstream watercourses converging at a single downstream sampling location (Figure 1). From the four sample sites, Goondaloo Creek (latitude 19.323687° S, longitude 146.762842° E) was selected for longitudinal investigation after it was determined to fulfil all the inclusion criteria (Supplementary Materials S1–S8). Water samples were collected weekly for over a year (February 2022–February 2023) whenever the creek contained water. In addition, the sampling frequency was increased opportunistically during periods of sustained rainfall. Daily rainfall data were collected from the Bureau of Meteorology, Climate Data Online [25]. A visual turbidity scale, with four qualitative categories, was used to assess collected water samples (Figure 2). The presence or absence of flowing water was recorded.

### 2.2. Sample Processing and Isolation of B. pseudomallei

One litre of groundwater from the study site was collected into sterile bottles. Ten replicate 100 µL volumes of sample water were inoculated onto Ashdown agar (ASH-A), incubated for two days at 37 °C in ambient air, and analysed for typical colonial morphology of *B. pseudomallei* to determine CFU/mL [28,29]. The sum of the characteristic *B. pseudomallei* colonies from all ten replicate plates was used to determine CFU/mL. The remaining water was filtered through 0.22 µm filters, and the paper was incubated in Ashdown broth (ASH-B) using similar incubation conditions. Approximately 10 µL of ASH-B was dilution-streaked onto ASH-A from each sample after one, two and five days of incubation. The remaining water samples were filtered and processed to enhance the sensitivity of *B. pseudomallei* detection. ASH-A and ASH-B media were selected in accordance with published consensus guidelines [1]. Comparative evaluations of selective media for *B. pseudomallei* are limited; consequently, Ashdown agar has remained the preferred selective medium in endemic regions. Although Ashdown agar is considered the standard and demonstrates specificity approaching 100%, it may inhibit the growth of mucoid and smooth colony variants *of B. pseudomallei*, as well as gentamicin-susceptible strains [30,31,32,33].

Quantification of *B. pseudomallei* colonies was determined by recognition of characteristic colony morphology and standard plate count methods. A subset of 10% or the square root of total colonies, if 10% was below 10 colonies, was collected to represent diversity [34,35,36]. A subset of suspect colonies was confirmed by Type III Secretion System (TTSS) qPCR modified from [5] (Supplementary Materials, Table S9). Briefly, the PCR reactions consisted of 1× GoTaq Colourless Master Mix (Promega, Madison, WI, USA), 0.4 µM of forward and reverse primers (Sigma-Aldrich, St. Louis, MO, USA), 0.256 µM of probe (Sigma-Aldrich), 50–100 ng of DNA, and molecular-grade H_2_O to 20 µL. Thermocycling conditions consisted of a 5 min initial denaturation at 95 °C, followed by 45 cycles of 15 s at 95 °C and 15 s at 59 °C. A single colony of bacteria was directly added to each PCR reaction (Colony PCR). A Mic qPCR Cycler (Bio Molecular Systems, Upper Coomera, QLD, Australia) (micPCR version 2.10.1) was used for all PCR reactions. Confirmed *B. pseudomallei* colonies were stored in 20% Glycerol Tryptic Soy Broth and frozen at −80 °C.

### 2.3. MLST PCR and Sequencing of B. pseudomallei Isolates

In the James Cook University Townsville PC3 facility, isolates were cultured on ASH-A, and DNA was extracted (Roche High Pure PCR Template kit (Roche Diagnostics, Mannheim, Germany)) from a single colony. Internal fragments of the seven MLST loci (*ace*, *gltB*, *gmhD*, *lepA*, *lipA*, *narK*, and *ndh*) were amplified using PubMLST primers and conditions (Supplementary Materials, Table S10) [37]. The reactions consisted of 1x GoTaq Colourless Master Mix (Promega), 0.4 µM of forward and reverse primers (Sigma-Aldrich), 50–100 ng of DNA and molecular-grade H_2_O to 30 µL. The thermocycling conditions for *ace, gltB, lipA, narK*, and *ndh* consisted of a 3 min initial denaturation at 95 °C, followed by 40 cycles of 30 s at 95 °C, 30 s at 62 °C, and 30 s at 72 °C, ending with a 10 min final elongation step at 72 °C. For *gmhD* and *lepA,* the annealing step was conducted at 59 °C after optimisation using a PCR temperature gradient. Amplification was performed on a CFX96 Real-Time System C1000 Touch Thermal Cycler (Bio-Rad Laboratories, Hercules, CA, USA), and products were verified by 1.5% agarose gel electrophoresis with a 100 bp HyperLadder (Meridian Bioscience, Eveleigh, NSW, Australia).

The seven MLST amplicons from each isolate were pooled, cleaned (Wizard SV Gel and PCR Clean-up System kit (Promega, Madison, WI, USA)), quantified (Qubit 3.0 Fluorometer (Thermo Fisher Scientific, Scoresby, VIC, Australia) and Qubit dsDNA HS Assay kit (Thermo Fisher Scientific, Scoresby, VIC, Australia)), and normalised (62 ng or approximately 200 fmol of DNA). Libraries were prepared with the ONT Native Barcoding kit (SQK-NBD114.24) (Oxford Nanopore Technologies, Littlemore, OX4 4DQ, United Kingdom), with up to 24 barcoded DNA samples pooled, and then run on a R10.4.1 FLO-MIN114 MinION flow cell. High-accuracy basecalling with barcode splitting was used; FASTQ files were exported per the Mk1C manual. Read quality and length were assessed with NanoPlot (version 1.42.0) [38,39]. For the ST assignment, approximately 8000 reads per isolate (more than 1000 reads per locus) were analysed via the Center for Genomic Epidemiology (CGE) webservice “https://cge.food.dtu.dk/services/MLST/ (accessed on 15 May 2024)” (v2.0.9 (11 May 2022)) using k-mer alignment (KMA; minimum depth ≥ 80×) against the *B. pseudomallei* pubMLST database (19 June 2023) [40,41]. KMA consensus sequences were also queried on pubMLST to confirm allele calls “https://pubmlst.org/organisms/burkholderia-pseudomallei (accessed 15 May 2024)” [42]. Twelve isolates underwent Illumina sequencing to validate the ONT sequencing results. Novel alleles and STs were submitted to PubMLST (Supplementary Materials, Tables S21 and S22).

### 2.4. Data Analysis

All descriptive statistics, measures of normality, and graphs were generated in GraphPad Prism (v10.0.1); all tests were nonparametric. The proportion of culture-positive samples was calculated with a Fraction of Total test. *B. pseudomallei* concentrations were compared between flowing versus non-flowing water by a Mann–Whitney test, and across turbidity categories by a Kruskal–Wallis test with Dunn’s Multiple Comparisons. Associations with climatic variables were assessed using Spearman’s Rank Correlation Coefficient and simple linear regression. Observed versus expected distributions of Goondaloo Creek STs were tested with a Chi-Square Test of Good Fitness test.

For phylogenetic analysis, concatenated MLST sequences from Townsville *B. pseudomallei* isolates, together with associated metadata spanning 1996–2023, were exported from pubMLST [42], merged with Goondaloo Creek STs, and rooted with Thailand ST10 [14]. Alignments were built with MAFFT (v7.505), and trees were inferred in IQ-TREE (v2.2.2.2) using ModelFinder with 1000 nonparametric bootstraps (nodes ≥ 80 shown) [14,43,44,45,46,47,48,49]. Trees were annotated and edited in iTOL (v6.9.1) [14,50].

## 3. Results

### 3.1. Annual Prevalence of B. pseudomallei in Creek Water

**Figure 3:**
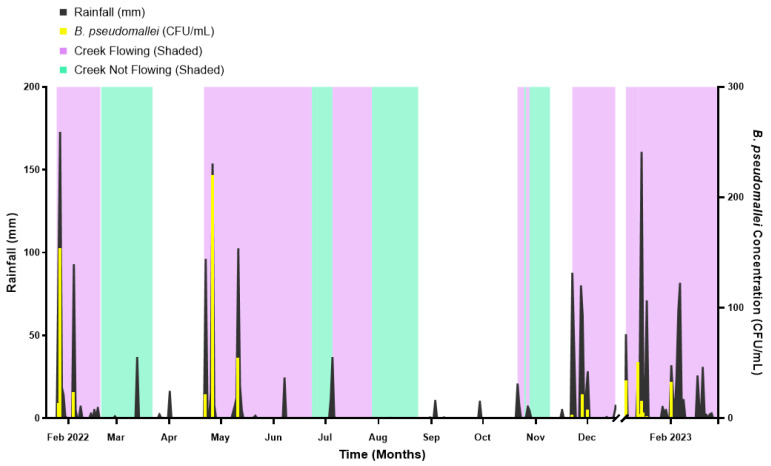
Longitudinal weekly and opportunistic sampling for *B. pseudomallei* concentration (CFU/mL) over time (months) against rainfall (mm) for Goondaloo Creek. Rainfall (mm) (black) is shown on the left y-axis (Supplementary Materials, Table S13). *B. pseudomallei* concentration (CFU/mL) (yellow) is shown on the right y-axis (Supplementary Materials, Table S11). Time (months) is shown on the x-axis. Purple shading represents periods when creek water was flowing, and green shading represents non-flowing water periods (Supplementary Materials, Table S14). No shading represents a period when the creek was dry (no water).

The prevalence of *B. pseudomallei* in creek water collected over a year from Goondaloo Creek is presented in Figure 3. Water samples collected from both weekly and opportunistic sampling revealed 18 out of 59 samples (30.5% [95% confidence interval (CI): 20.25 to 43.15]) were culture-positive for *B*. *pseudomallei* (Supplementary Materials, Table S11). TTSS qPCR was used for confirmation of selected colonies (Supplementary Materials, Table S12). Of the 669 presumptive *B. pseudomallei* colonies observed on ASH-A, 66 (approximately 10%) were selected and subsequently confirmed as *B. pseudomallei* by TTSS-targeted qPCR.

### 3.2. Prevalence and Concentration of B. pseudomallei Correlates with Extent of Rainfall

**Figure 4:**
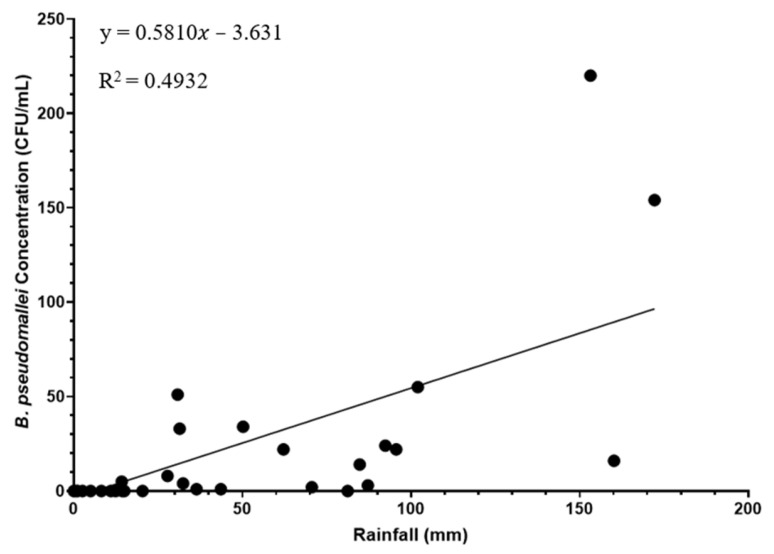
Concentration of *B. pseudomallei* (CFU/mL) in creek water during different volumes of rainfall (mm). A simple linear regression model was fitted to the data; the line represents the best fit, the equation of the line and R^2^ values are included on the graph (Supplementary Materials, Tables S11 and S13).

*Burkholderia pseudomallei* was isolated from creek water only during the two days following heavy rainfall (Figure 3). The concentration of *B. pseudomallei* was strongly correlated with rainfall (*p* ≤ 0.0001; r = 0.8141; 95% CI: 0.7010–0.8872) (Figure 4). However, the total extent of rainfall was only a weak predictor of *B. pseudomallei* concentration (R^2^ = 0.4932; F(1, 57) = 55.47; *p* ≤ 0.0001), with a fitted regression model of y = 0.5810x − 3.631. Notably, *B. pseudomallei* was not detected until cumulative rainfall reached approximately 30 mm, suggesting the presence of a rainfall threshold associated with mobilisation or persistence (Supplementary Materials, Table S13).

### 3.3. Detection of B. pseudomallei Correlates with Water Flow and Water Turbidity

**Figure 5:**
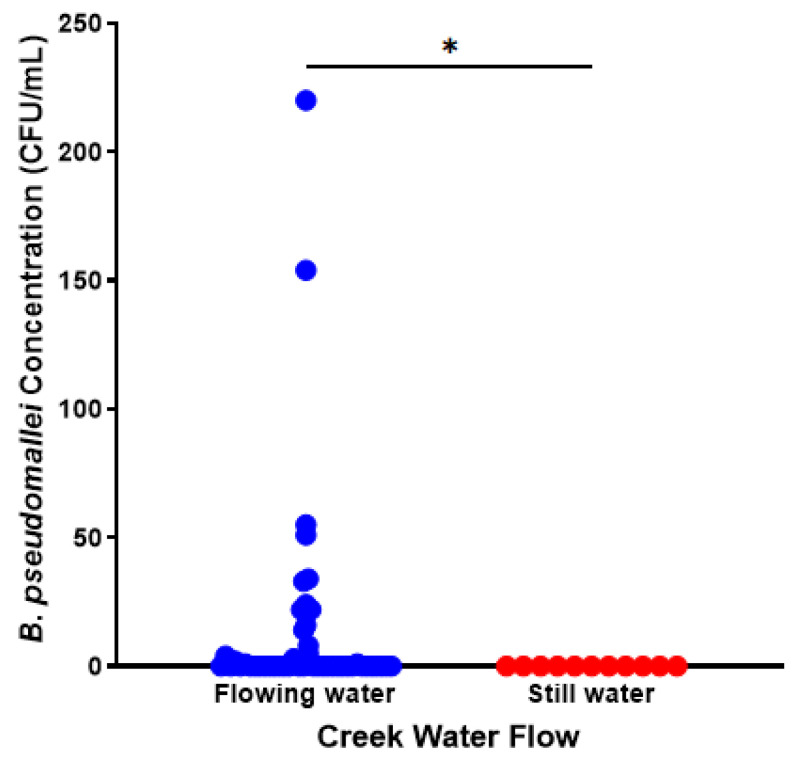
*B. pseudomallei* is found at higher concentrations in flowing water rather than still water. Flowing water is shown as blue (*n* = 48), and still water is shown as red (*n* = 11). A statistically significant comparison is represented by * (*p* ≤ 0.05) (Supplementary Materials, Tables S11 and S14).

**Figure 6:**
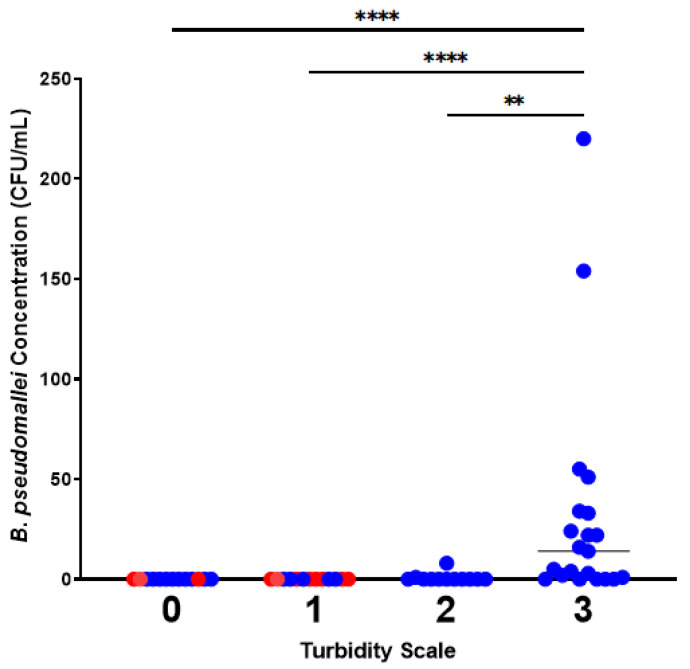
The highest *B. pseudomallei* concentration (CFU/mL) is in turbid water. Data representing flowing water and still water are coloured blue (*n* = 48) and red (*n* = 11), respectively. Statistically significant comparisons are represented by ** (*p* ≤ 0.01), *** (*p* ≤ 0.001), and **** (*p* ≤ 0.0001) (Supplementary Materials, Tables S11 and S15).

*Burkholderia pseudomallei* was exclusively isolated from flowing and turbid creek water compared with water that was not flowing and clear (Figure 5 and Figure 6) (Supplementary Materials, Tables S11 and S14). *B. pseudomallei* was only detected in turbidity-level-two or -three water (Figure 6 and Supplementary Materials, Table S15). Turbidity-level-two water yielded concentrations below 10 CFU/mL, while level-three water yielded concentrations up to 220 CFU/mL (level zero vs. three (*p* ≤ 0.0001), level one vs. three (*p* ≤ 0.0001), and level two vs. three (*p* = 0.0011)). These findings suggest that turbidity is a strong indicator of *B. pseudomallei* presence in water and may reflect the contribution of rainfall-driven soil erosion and surface runoff processes to the mobilisation of *B. pseudomallei* into aquatic environments.

### 3.4. Genomic Epidemiology of Goondaloo Creek Isolates

A total of 50 *B. pseudomallei* isolates were sequenced using ONT sequencing, two *B. pseudomallei* controls (ST276 and ST814) and 48 Goondaloo Creek *B. pseudomallei* isolates. Creek isolates chosen for sequencing were representative of STs present in water samples and across all sampling timepoints (Supplementary Materials S16–S18 and S20).

**Figure 7:**
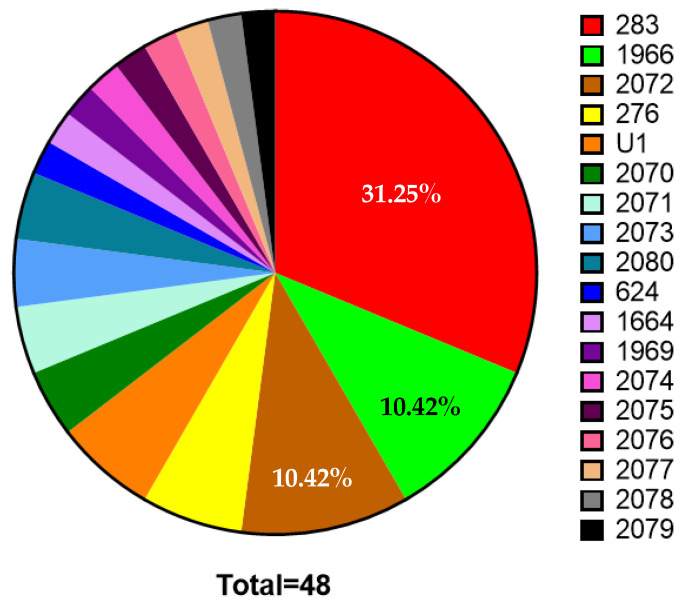
Diversity and abundance of Goondaloo Creek STs. Identified STs are included in the key, listed from most to least abundant. Refer to Supplementary Materials, Table S23, for all complete number and percentage values for each ST.

Targeted ONT sequencing of 48 isolates resolved 18 STs, comprising 12 novel STs and six previously reported in pubMLST. Two novel alleles were detected: *lipA* (single-nucleotide variant; accepted by pubMLST) and *lepA* (3 bp insertion; not accepted). Consequently, 45/48 isolates received complete ST assignments, while three clonally related isolates remained incomplete due to the novel *lepA* allele. Both alleles were confirmed by Illumina WGS (Supplementary Materials, Tables S19 and S22). The most frequent STs were ST283 (15/48, 31.3%), ST1966 (5/48, 10.4%), and ST2072 (5/48, 10.4%) (Figure 7 and Supplementary Materials, Table S23). The ST frequencies deviated from a uniform distribution (expected 5.556% per ST; *p* ≤ 0.0001).

**Figure 8:**
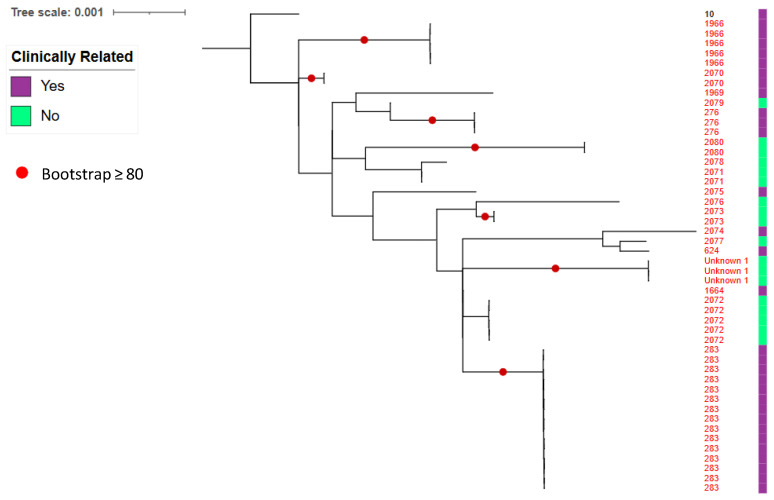
Phylogenetic tree of Goondaloo Creek *B. pseudomallei* STs. Goondaloo Creek *B. pseudomallei* STs (red numbers), PubMLST matches to clinical human and animal case STs (purple) and lack of matches (green) are shown to the right of the phylogenetic tree. ‘Unknown 1’ refers to three of the sequenced Goondaloo Creek isolates that have a novel *lepA* and cannot be assigned an ST. A bootstrapping value greater than 80% is depicted by a red circle on the phylogenetic tree. The tree is rooted at ST10 (black number).

Nine STs (ST276, ST283, ST624, ST1664, ST1966, ST1969, ST2070, ST2074, and ST2075) matched STs previously recorded from human and/or animal cases in pubMLST (Figure 8 and Supplementary Materials, Table S21). ST283 had the most clinical links (five human and five animal submissions).

**Figure 9:**
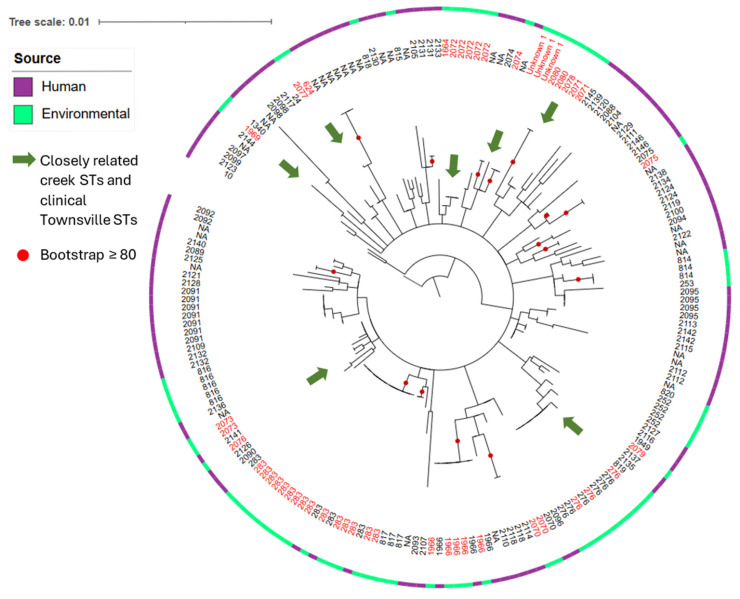
Phylogenetic tree of Townsville STs and Goondaloo Creek STs. From outside in, PubMLST matches to clinical human STs (purple) and environmental STs (green), Townsville STs (black numbers) and Goondaloo Creek STs (red numbers), and clades containing both Goondaloo Creek STs and Townsville clinical STs (green arrow). A bootstrapping value greater than 80% is depicted by a red circle. The tree is rooted at ST10 (black number).

Concordance with Townsville clinical epidemiology was evident: Goondaloo Creek ST1966, ST276, ST283, ST2070, ST2074, and ST2075 matched prior Townsville human clinical STs (Figure 9), and ST276 also matched previously detected environmental ST276 in Townsville. Additional creek STs (ST624, ST1969, ST2071–ST2080, and Unknown 1) were grouped within clades containing Townsville clinical STs, although these placements lacked bootstrap support.

## 4. Discussion

Groundwater and groundwater–surface water interfaces are increasingly recognised as important reservoirs and exposure pathways for *B. pseudomallei* in northern Australia [5,6,7,8,9]. This study provides longitudinal evidence that a seasonal creek functions as a hydrologically triggered conduit, rather than a static reservoir, for the mobilisation of *B*. *pseudomallei* into surface water following rainfall.

Isolation of *B. pseudomallei* was consistently preceded by rainfall events of approximately ≥30 mm and largely confined to periods of active flow and elevated turbidity. Concentrations increased with rainfall intensity and persisted for up to two days following heavy rainfall, extending during sustained precipitation. These patterns are consistent with sediment-linked mobilisation via lateral subsurface flow, erosion, and suspended sediment transport observed in other hydrologic systems [10,51,52]. In contrast, stagnant and typically clear creek conditions were associated with little or no detection, indicating that short-term mobilisation, rather than in situ persistence, governs presence in this system. The strong association between turbidity and *B. pseudomallei* concentration supports a sediment-mediated transport mechanism. Similar relationships have been reported in Laos, where rivers act as carriers for *B*. *pseudomallei* following rainfall [10,14]. Collectively, these findings suggest that seasonal creeks generate brief but potentially high-risk exposure windows immediately following rainfall events. Exposure appears to be associated with eroded soils containing *B. pseudomallei*, which contaminate groundwater and seasonal creeks following periods of high rainfall.

Genomic analysis identified substantial strain diversity, including multiple sequence types previously associated with human and animal infection. Several creek isolates matched Townsville clinical sequence types, including ST283 and ST276, supporting the clinical relevance of organisms mobilised through this system [44,53]. Clustering within established Townsville clades indicates longstanding local establishment. While MLST resolution was sufficient to demonstrate lineage overlap, whole-genome sequencing will be required to more precisely resolve transmission pathways and dispersal dynamics.

The short detection window contrasts with studies reporting broader wet-season persistence in drains and depositional environments [8,14], likely reflecting hydrologic context. Flowing creek systems may rapidly mobilise and export organisms downstream, whereas slower-moving or depositional systems permit longer persistence. Washed-out organisms may subsequently accumulate in downstream habitats, extending exposure risk beyond the immediate site.

These findings have important public health implications. In Darwin, increased hydrologic connectivity during La Niña conditions was hypothesised to facilitate expansion of specific *B. pseudomallei* lineages [9]. In Townsville, shifts in sequence type prevalence over time have been documented without a defined mechanism [44,53]. Groundwater-mediated transport through connected surface-water networks represents a plausible contributor to the redistribution of established local lineages across catchments, particularly during extreme rainfall events.

The sampling year was characterised by frequent environmental detection *of B. pseudomallei* (this study), alongside elevated regional melioidosis notifications during the same period [24]. Although direct attribution cannot be made, the concordance between rainfall intensity, environmental detection, and case incidence mirrors established associations observed in Darwin and Townsville [22,23] and aligns with outbreak reports following extreme rainfall elsewhere in Australia [2,54,55].

From a surveillance perspective, groundwater sampling from urban creeks provides an efficient approach for detecting *B. pseudomallei* across broad catchments, complementing soil-based investigations that are labour-intensive and spatially heterogeneous [19,56]. Incorporating rainfall thresholds, turbidity metrics, and hydrologic connectivity into monitoring frameworks may improve identification of high-risk exposure windows, particularly under projected climate scenarios characterised by increasing rainfall intensity and flooding [26].

Several limitations warrant consideration. This study focused on a single seasonal creek, and applicability to other catchments with differing geomorphology and aquifer connectivity remains uncertain. Weekly sampling may not have captured very short-lived mobilisation events, and rainfall data were derived from a station approximately nine kilometres from the site. Genomic analyses were limited to MLST. Future studies should integrate higher-frequency sampling, on-site hydrometeorological monitoring, sediment characterisation, and whole-genome sequencing across multiple catchments.

In North Queensland, where groundwater discharge, climatic variability, and urban expansion intersect, seasonal creeks represent dynamic interfaces capable of rapidly mobilising clinically relevant *B. pseudomallei.* As climate projections indicate increasing rainfall intensity and flash flooding [26], rainfall-triggered mobilisation events are likely to become more frequent. Integrating hydrologic monitoring, genomic epidemiology, and environmental surveillance will be critical for defining spatiotemporal risk and informing targeted public health interventions in a changing climate.

## Data Availability

The datasets used and/or analysed during the current study are available from the corresponding author upon reasonable request.

## Supplementary Materials

The following supporting information can be downloaded at https://www.mdpi.com/article/10.3390/pathogens15030276/s1, Supplementary Materials S1: Selection of Location for Temporal Study.Supplementary Materials S2: Surface Groundwater Sampling Locations. Supplementary Materials S3: Inclusion Criteria. Supplementary Materials S4: Temporal Study Site. Supplementary Materials S5: *B. pseudomallei* Concentrations for Each Sampling Location. Supplementary Materials S6: Roundtrip Travel Times. Supplementary Materials S7: Sampling Locations and their Relative Position to Waterways. Supplementary Materials S8: Sampling Location Inclusion Criteria for Longitudinal Study. Supplementary Materials S9: TTSS Primer and Probe Sequences. Supplementary Materials S10: MLST Primer Sequences. Supplementary Materials S11: Temporal Study Direct Plate Count Raw Data. Supplementary Materials S12: Temporal Study TTSS PCR Raw Data. Supplementary Materials S13: Temporal Study Daily Rainfall Raw Data. Supplementary Materials S14: Temporal Study Flowing Water Raw Data. Supplementary Materials S15: Temporal Study Visual Turbidity Raw Data. Supplementary Materials S16: Validation of ONT Sequencing using Illumina WGS. Supplementary Materials S17: Comparison of Targeted ONT and Illumina WGS. Supplementary Materials S18: Targeted ONT Sequencing ST Determination of *B. pseudomallei* Isolates Compared to Illumina WGS. Supplementary Materials S19: Sequence Similarity between ONT and Illumina Sequences. Supplementary Materials S20: PubMLST Sequence Submission. Supplementary Materials S21: Creek Isolate IDs, STs, and PubMLST IDs. Supplementary Materials S22: DNA Sequences for lipA and lepA Alleles. Supplementary Materials S23: Number and Percentage Totals for Sequenced Creek Isolates.

## Abbreviations

The following abbreviations are used in this manuscript:

*ace*	Acetyl Coenzyme A Reductase
ASH-A	Ashdown Agar
ASH-B	Ashdown Broth
BOM	Bureau of Meteorology
bp	Base Pairs
CFU	Colony-Forming Unit
CGE	Center for Genomic Epidemiology
CSIRO	Commonwealth Scientific and Industrial Research Organisation
DNA	Deoxyribonucleic Acid
*gltB*	Glutamate Synthase
GTSB	Glycerol Tryptic Soy Broth
*gmhD*	ADP Glycerol-Mannoheptose Epimerase
JCU	James Cook University
KMA	k-mer Alignment
*lepA*	GTP-Binding Elongation Factor
*lipA*	Lipoic Acid Synthetase
MLST	Multi-Locus Sequence Typing
*narK*	Nitrite Extrusion Protein
*ndh*	NADH Dehydrogenase
ONT	Oxford Nanopore Technology
PNG	Papua New Guinea
PCR	Polymerase Chain Reaction
qPCR	Quantitative Real-Time PCR
ST	Sequence Type
TTSS	Type III Secretion System
WGS	Whole-Genome Sequencing
