# Supplementary material for: Rainfall-Driven Mobilisation of Clinically Relevant Burkholderia pseudomallei in a Groundwater-Connected Urban Creek, Northern Australia
Source: Pathogens. 2026 Mar 3;15(3):276. doi: 10.3390/pathogens15030276 (PMC13029273; doi:10.3390/pathogens15030276)
Supplement: Supplementary file 1 [file pathogens-15-00276-s001.zip › pathogens-4174298-supplementary.pdf]

Janssen-Groesbeek et al. https://doi.org/10.3390/xxxxx

Supplementary Materials S1-S23

S1. Selection of Location for Temporal Study

Previous studies conducted in Townsville showed *B. pseudomallei* was present at the following sites across the Townsville region: Mt Stuart, Mt Louisa, and Castle Hill [1,2]. Goondaloo Creek contains water that flows off Mt Stuart after rainfall and is located at James Cook University (JCU). These four endemic sites were selected and assessed on a single occasion after heavy rain, for use as a sentinel site location for a longitudinal study (Figure S2). Samples were collected at the following GPS points (determined by ArcGIS Field Maps; https://www.arcgis.com/apps/mapviewer/) for each site: Mt Stuart (latitude 19.344022°S, longitude 146.781673°E), Mt Louisa (latitude 19.271080°S, longitude 146.724317°E), Castle Hill (latitude 19.259098°S, longitude 146.806614°E), and Goondaloo Creek (latitude 19.323687°S, longitude 146.762842°E). Exact locations were selected at time of sample collection based on presence of collectible flowing surface groundwater. The criteria for inclusion were presence of high concentrations (> 14 CFU/mL, mean concentration of *B. pseudomallei* in surface groundwater surrounding Castle Hill previously) of *B. pseudomallei*, proximity of sampling site to JCU (≤ 30min roundtrip, where a roundtrip is travel to and from the location), and broad area representation of collected water sample (multiple watercourses converging into single site) (Figure S3) [1]. Samples were collected within 24 hours of significant rainfall and were collected, processed, and quantified as described in the Materials and Methods.

S2. Surface Groundwater Sampling Locations

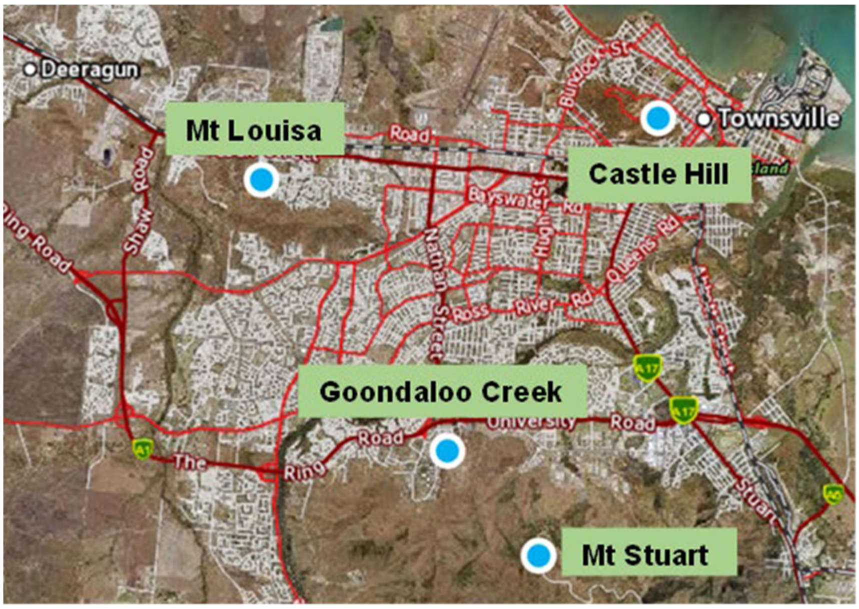

Figure S2 - Surface groundwater sampling locations across Townsville. Map modified from Queensland Globe. Map copyright © The State of Queensland (Department of Natural Resources and Mines, Manufacturing and Regional and Rural Development) 2025. Map licensing © CNES reproduced under license from Airbus DS, all rights reserved © 21AT © Earth-i, all rights reserved, © Planet Labs PBC, 2025.

S3. Inclusion Criteria

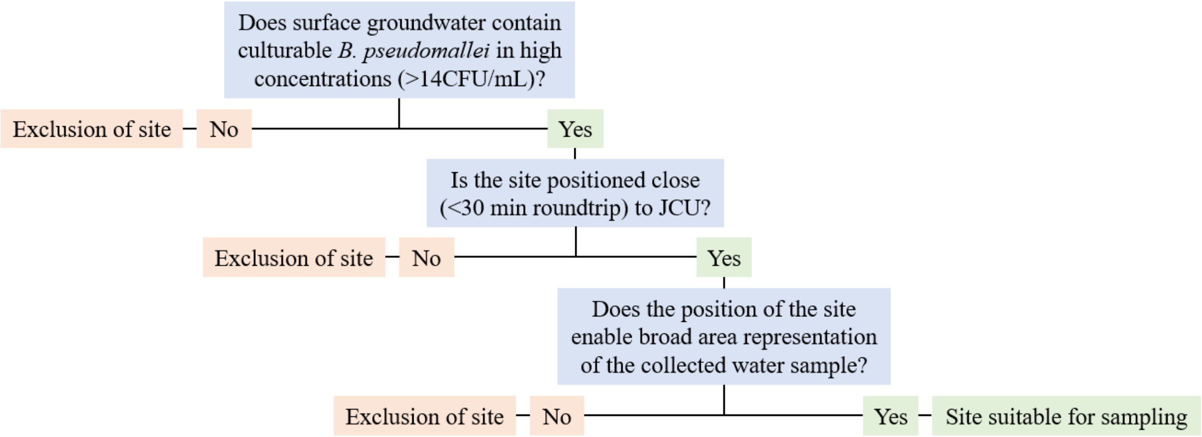

Figure S3 - Inclusion criteria applied to surface groundwater sampling sites for selection of a longitudinal sampling site as described in Section S1.

S4. Temporal Study Site

Burkholderia pseudomallei was detected in three of four locations, including Mt Louisa, Mt Stuart, and Castle Hill (Figure S5). Goondaloo Creek (Mt Stuart) was closest to James Cook University, Townsville campus and most easily accessed (Table S6). Goondaloo Creek was a drain catchment for surface groundwater that flowed off Mt Stuart, representing a broad area from which surface groundwater originated from (Figure 1 and Figure S7). Goondaloo Creek fulfilled all inclusion criteria described in Section S1 and was selected for longitudinal sampling (Table S8).

S5. B. pseudomallei Concentrations for Each Sampling Location

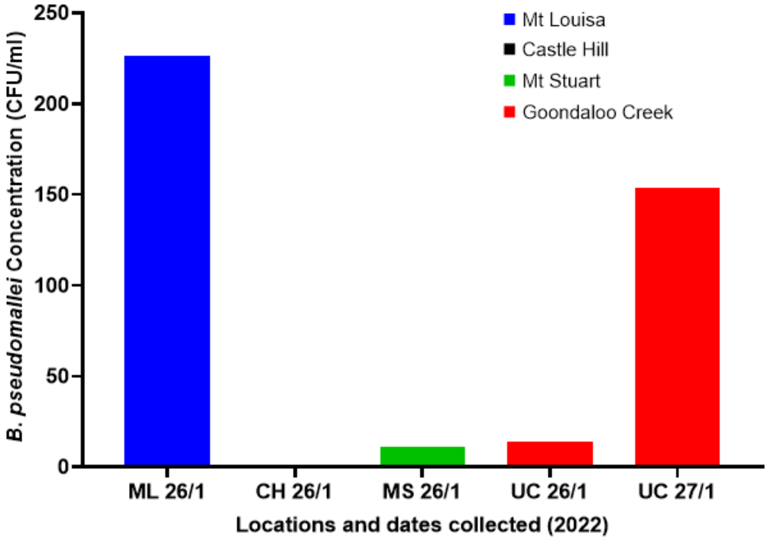

Figure S5 - Concentration of *B. pseudomallei* (CFU/mL) from each sampling location. Sampling locations were Mt Louisa (ML sampled on 26th January 2022), Castle Hill (CH sampled 26th January 2022), Mt Stuart (MS sampled on 26th January 2022), and Goondaloo Creek (UC sampled on 26th and 27th January 2022) (Table S11)

S6. Roundtrip Travel Times

Table S6 – Roundtrip travel time to each of the initial sampling locations.

Initial Sampling Locations	Roundtrip Travel Time by Car (minutes)	Roundtrip Travel Time by Walking (minutes)
Mt Stuart	56	NA
Mt Louisa	30	NA
Castle Hill	50	NA
Goondaloo Creek	4	12

S7. Sampling Locations and their Relative Position to Waterways

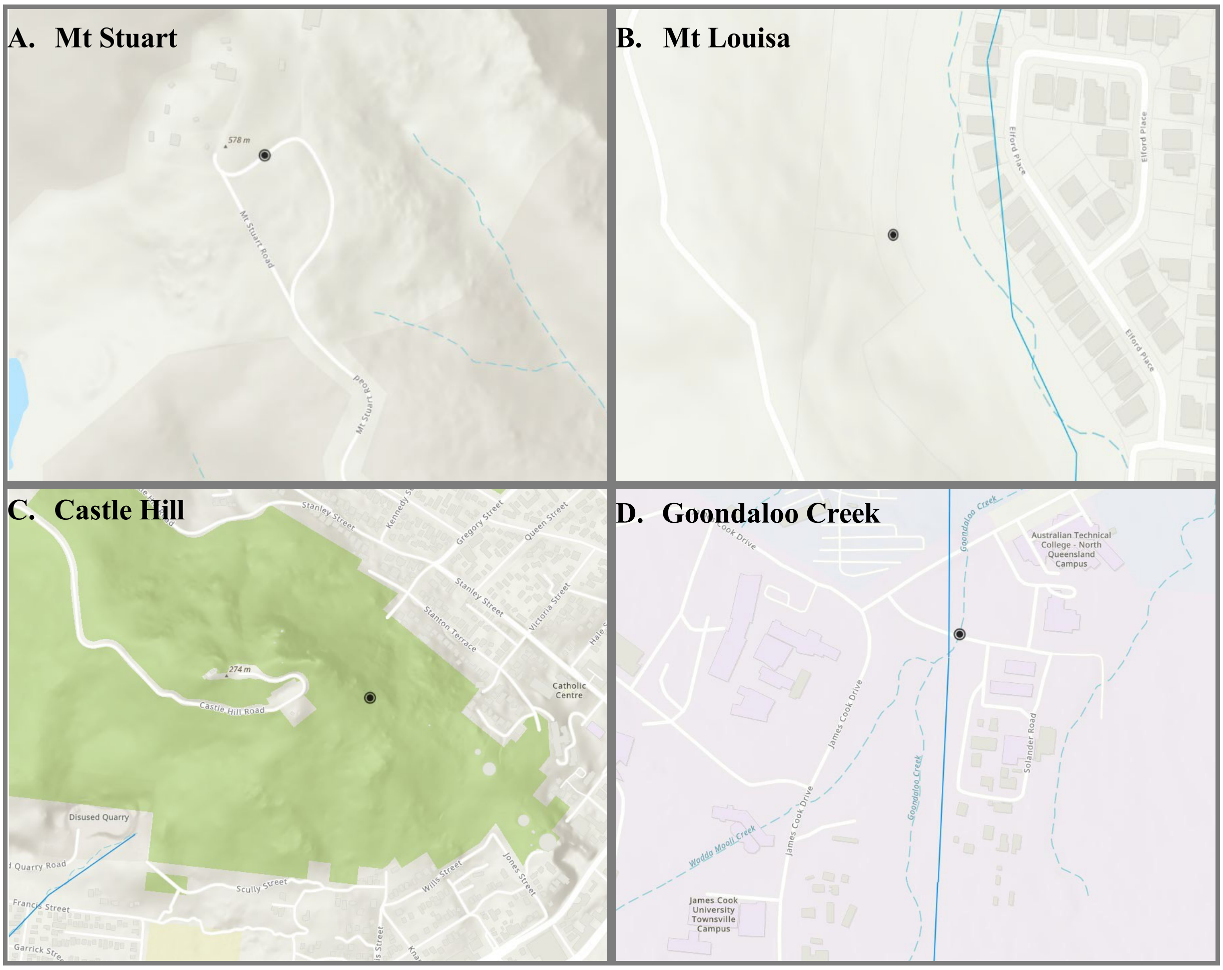

Figure S7 – Initial sampling locations and their position relative to waterways to determine if collected water samples will represent a broad area. Major waterways are depicted as solid blue lines and minor waterways are shown as dotted blue lines. Initial sampling locations are shown as black circles. Maps created in ArcGIS Online. Map copyright Queensland Wetlands Program in the Department of Environment and Science, Sources: Esri, TomTom, Garmin, FAO, NOAA, USGS, ©.

S8. Sampling Location Inclusion Criteria for Longitudinal Study

Table S8 - Longitudinal study inclusion criteria by each sampling location.

Location	Inclusion Criteria (tick represents satisfied criteria)		
	High concentration of *B. pseudomallei* (>14 CFU/mL)	Close proximity to JCU (< 30min roundtrip)	Broad area representation of water sample
Mt Louisa	✓	✓	
Castle Hill			
Mt Stuart			
Goondaloo Creek	✓	✓	✓

S9. TTSS Primer and Probe Sequences

Table S9 – TTSS primer and probe sequences.

**Primer/Probe**	**Sequence (5' – 3')**
BpTT4176-F	CGTCTCTATACTGTCGAGCAATCG
BpTT4290-R	CGTGCACACCGGTCAGTATC
BpTT4208-P	FAM-CCGGAATCTGGATCACCACCACTTTCC-BHQ

S10. MLST Primer Sequences

Table S10 – MLST primer sequences.

**Primer**	**Sequence (5' – 3')**
ace-up	CGGCGCTTCTCAAAACGATA
ace-dn	GAATCGCCTTCACCATGTC
gltB-up	ACGCTCGCGATCGCGATGAA
gltB-dn	TTCAGCACGAGCGTCTGCTG
gmhD-up	GCAGTTCCTGTATGCGTC
gmhD-dn	GAAGCACTGGTACTTGCC
lepA-up	CATATTCGCAATTTCTCGATC
lepA-dn	CACGAGCATCACGACGCCG
lipA-up	GGCACCGCGACGTTCATG
lipA-dn	GACCATCAGGCCCGATTTCG
narK-up	CTACTCGTGCGCTGGGAT
narK-dn	GACGATGAACGGCACCCAC
ndh-up	AGTCGCGACGTTCTACAC
ndh-dn	CGAGTTGCAGACGAGATA

S11. Temporal Study Direct Plate Count Raw Data

Table S11 - Direct plate count results from one-year longitudinal study of Goondaloo Creek.

Sample Date	Total CFU/mL	*Bps* CFU/mL	Sample Date	Total CFU/mL	*Bps* CFU/mL
26.1.22	822	14	21.10.22	1086	0
27.1.22	1593	154	26.10.22.1	141	0
2.2.22	29	0	26.10.22.2	148	0
3.2.22	273	0	26.10.22.3	393	0
4.2.22	343	24	2.11.22	132	0
9.2.22	20	0	22.11.22	422	3
16.2.22	2	0	23.11.22	992	1
23.2.22	343	0	28.11.22	556	22
2.3.22	112	0	30.11.22	431	0
9.3.22	2	0	1.12.22	149	8
16.3.22	7	0	7.12.22	51	0
22.4.22	1413	22	12.12.22	44	0
26.4.22	983	220	6.1.23	212	34
28.4.22	197	0	11.1.23	62	0
4.5.22	58	0	13.1.23	421	51
10.5.22	72	0	14.1.23	255	4
11.5.22	236	55	15.1.23	417	16
18.5.22	38	0	16.1.23	410	5
25.5.22	23	0	17.1.23	912	0
1.6.22	48	0	18.1.23	194	2
8.6.22	44	0	19.1.23	156	0
15.6.22	32	0	20.1.23	44	0
22.6.22	220	0	25.1.23	160	0
29.6.22	16	0	1.2.23	305	33
5.7.22	196	1	6.2.23	95	0
13.7.22	46	0	8.2.23	88	0
20.7.22	36	0	15.2.23	24	0
27.7.22	66	0	17.2.23	24	0
3.8.22	7	0	20.2.23	363	0
10.8.22	4	0	22.2.23	21	0
17.8.22	5	0			

S12. Temporal Study TTSS PCR Raw Data

Table S12 - TTSS PCR results for selected colonies from direct plate count plates and broth plates created from Goondaloo Creek water samples.

Sample Date	PCR Result	Sample Date	PCR Result	Sample Date	PCR Result	Sample Date	PCR Result
27.7.22-1	Positive	21.10.22-5	Negative	27.1.22-19	Positive	17.2.23-1	Negative
5.7.22-1	Positive	21.10.22-6	Negative	5.7.22-1	Positive	18.1.23-2	Negative
27.1.22-12	Positive	22.11.22-3	Negative	13.1.23-1	Positive	18.1.23-1	Positive
21.10.22	Negative	22.11.22-4	Negative	22.11.22-7	Negative	19.1.23-1	Negative
26.10.22-3	Negative	23.11.22-2	Negative	4.2.22-9	Positive	20.1.23-1	Negative
26.10.22-2	Negative	26.10.22-4	Negative	4.2.22-10	Positive	23.11.22-3	Negative
27.1.22-12	Positive	6.1.23-1	Positive	1.12.22-6	Negative	25.1.23-1	Negative
22.11.22-5	Negative	6.1.23-2	Positive	1.2.23-6	Negative	27.7.22-1	Positive
22.11.22-6	Negative	6.1.23-3	Positive	1.2.23-1	Positive	28.11.22-7	Negative
1.12.22-1	Positive	6.1.23-4	Positive	1.2.23-2	Positive	30.11.22-1	Negative
1.12.22-2	Negative	6.1.23-5	Positive	1.2.23-3	Positive	6.1.23-17	Negative
1.12.22-3	Positive	6.1.23-6	Positive	1.2.23-4	Positive	7.12.22-1	Negative
1.12.22-4	Negative	6.1.23-7	Positive	1.2.23-5	Positive	8.2.23-1	Negative
1.12.22-5	Positive	6.1.23-8	Positive	11.1.23	Negative	15.2.23-1	Negative
21.10.22-1	Negative	6.1.23-9	Positive	12.12.22	Negative	20.2.23-1	Negative
21.10.22-2	Negative	6.1.23-10	Positive	13.1.23-4	Negative	22.2.23-1	Negative
21.10.22-3	Negative	6.1.23-11	Positive	13.1.23-2	Positive	4.2.22-11	Positive
21.10.22-4	Negative	6.1.23-12	Positive	13.1.23-3	Positive	6.2.23-1	Negative
22.11.22-1	Negative	6.1.23-13	Positive	14.1.23-3	Negative	27.1.22-20	Positive
22.11.22-2	Positive	6.1.23-14	Positive	14.1.23-1	Positive	27.1.22-21	Positive
23.11.22-1	Positive	6.1.23-15	Positive	14.1.23-2	Positive	27.1.22-22	Positive
26.10.22-1	Negative	6.1.23-16	Positive	15.1.23-5	Negative	27.1.22-23	Positive
26.10.22-2	Negative	27.1.22-11	Positive	15.1.23-1	Positive	27.1.22-24	Positive
26.10.22-3	Negative	27.1.22-12	Positive	15.1.23-2	Positive		
28.11.22-1	Positive	27.1.22-13	Positive	15.1.23-3	Positive		
28.11.22-2	Positive	27.1.22-14	Positive	15.1.23-4	Positive		
28.11.22-3	Negative	27.1.22-15	Positive	16.1.23-3	Negative		
28.11.22-4	Positive	27.1.22-16	Positive	16.1.23-1	Positive		
28.11.22-5	Positive	27.1.22-17	Positive	16.1.23-2	Positive		
28.11.22-6	Positive	27.1.22-18	Positive	17.1.23-1	Negative		

S13. Temporal Study Daily Rainfall Raw Data

Table S13 - Daily rainfall (mm) data from January 2022 to February 2023 for Townsville Airport weather station (station number 032040, latitude: 19.25° S, longitude:146.77° E).

Day	Jan 2022	Feb	Mar	Apr	May	Jun	Jul	Aug	Sep	Oct	Nov	Dec	Jan 2023	Feb
**1**	0	0	0	16	0	0	0	0	0	0	0	27.8	9	31.4
**2**	0	0	0	0.2	0	0	0	0	0.2	0	0.2	0	12	13.4
**3**	22.4	0	0	0	0.2	0	0.4	0	10.4	0	0	0	0	2.6
**4**	0	92.4	0	0.2	0	0	12	0	0	0	0	0	0	25
**5**	0	5.2	0	0.2	0	0	36.4	0	0	0	0	0	0.4	64.8
**6**	0	0	0	0	0	0	1	0	0	0	0	0	50.2	81.2
**7**	16.8	0	0	0	0	24	0	0	0	0	0	0	0	2.2
**8**	0	7	0	0	3.8	0	0.2	0.4	0.6	0	0	0	0.2	11
**9**	0	0	0	0.2	7.8	0	0	0	0	0	0	0	0	0
**10**	0.2	0	0	0	12.4	0	0	0	0	0	0	0	0	0
**11**	2	0	0	0	102	0	0	0	0	0	0	0	0	0
**12**	0.2	0	0	0	19.6	0	0	0	0	0	0	0.8	0	0
**13**	0	0.2	0	0	5	0	0	0	0	0	0	0	30.8	0
**14**	0	2.8	36.4	0	0	0	0	0	0	0	0	0	32.4	0
**15**	0	0	0.2	0	0	0	0	0	0	0	0	0	160.2	0
**16**	0	5	0	0	0	0	0	0	0	0	5	0.8	14.2	25.2
**17**	0	0.2	0	0	0	0	0	0	0	0		5.6	14.4	8.2
**18**	0	6.2	0	0	0	0	0	0	0			8.2	70.6	1.2
**19**	5.6	0	0	0	0	0	0	0	0	0	0	2.4	0.8	30.4
**20**	0	0	0	0	0.6	0	0	0	0	0.2	0	0.2	0	2.6
**21**	0	0	0	0	1.6	0	0	0	0	20.4	0.2	0	0	2.2
**22**	0	0	0	95.6	0	0	0	0	0	8.4	87.2	0	0	0
**23**	0	0	0	4	0	0	0	0	0	0	43.6	0	0	2.4
**24**		0	0	0.2	0	0	0	0	0	0	0	0	0	3
**25**	0	0	0	15.6	0	0	0	0	0	0	0	25.2	0	0
**26**	84.8	0	2.4	153.2	0	0	0	0	0	1	0	1	0	0
**27**	172.2	0	0	8	0	0	0	0	0	7	79.6	0.6	6.8	0
**28**	18.6	1.2	0.4	0	0	0	0	0	0	4.6	62.2	0	3.6	0
**29**	14		0.2	0	0	0	0	0	10	0	0.2	0	5	
**30**	1.4		0	0	0	0	0	0	0	0	15	0	0	
**31**	0		0		0		0	0.6		0		0	0	

S14. Temporal Study Flowing Water Raw Data

Table S14 - Flowing water data (yes/no) for collected water samples from Goondaloo Creek.

**Sample Date**	**Flowing Data**	**Sample Date**	**Flowing Data**
26.1.22	Yes	21.10.22	Yes
27.1.22	Yes	26.10.22	Yes
2.2.22	Yes	2.11.22	No
3.2.22	Yes	22.11.22	Yes
4.2.22	Yes	23.11.22	Yes
9.2.22	Yes	28.11.22	Yes
16.2.22	Yes	30.11.22	Yes
23.2.22	No	1.12.22	Yes
2.3.22	No	7.12.22	Yes
9.3.22	No	12.12.22	Yes
16.3.22	No	6.1.23	Yes
22.4.22	Yes	11.1.23	Yes
26.4.22	Yes	13.1.23	Yes
28.4.22	Yes	14.1.23	Yes
4.5.22	Yes	15.1.23	Yes
10.5.22	Yes	16.1.23	Yes
11.5.22	Yes	17.1.23	Yes
18.5.22	Yes	18.1.23	Yes
25.5.22	Yes	19.1.23	Yes
1.6.22	Yes	20.1.23	Yes
8.6.22	Yes	25.1.23	Yes
15.6.22	Yes	1.2.23	Yes
22.6.22	No	6.2.23	Yes
29.6.22	No	8.2.23	Yes
5.7.22	Yes	15.2.23	Yes
13.7.22	Yes	17.2.23	Yes
20.7.22	Yes	20.2.23	Yes
27.7.22	No	22.2.23	Yes
3.8.22	No		
10.8.22	No		
17.8.22	No		

S15. Temporal Study Visual Turbidity Raw Data

Table S15 - Visual turbidity data (0-3) for collected water samples from Goondaloo Creek.

Sample Date	Visual Turbidity	Sample Date	Visual Turbidity
26.1.22	3	21.10.22	3
27.1.22	3	26.10.22	3
2.2.22	2	2.11.22	1
3.2.22	2	22.11.22	3
4.2.22	3	23.11.22	2
9.2.22	2	28.11.22	3
16.2.22	0	30.11.22	2
23.2.22	0	1.12.22	2
2.3.22	1	7.12.22	0
9.3.22	1	12.12.22	0
16.3.22	1	6.1.23	3
22.4.22	3	11.1.23	2
26.4.22	3	13.1.23	3
28.4.22	3	14.1.23	3
4.5.22	0	15.1.23	3
10.5.22	2	16.1.23	3
11.5.22	3	17.1.23	3
18.5.22	2	18.1.23	3
25.5.22	0	19.1.23	2
1.6.22	0	20.1.23	2
8.6.22	0	25.1.23	1
15.6.22	0	1.2.23	3
22.6.22	0	6.2.23	3
29.6.22	1	8.2.23	2
5.7.22	3	15.2.23	1
13.7.22	0	17.2.23	1
20.7.22	0	20.2.23	1
27.7.22	0	22.2.23	1
3.8.22	1		
10.8.22	1		
17.8.22	1		

S16. Validation of ONT Sequencing using Illumina WGS

From the 48 Goondaloo Creek *B. pseudomallei* ONT sequenced isolates, 12 were selected for Illumina Whole Genome Sequencing (WGS) (Australian Genome Research Facility (AGRF)). Selection criteria included isolates that had novel profiles or alleles identified from Oxford Nanopore Technology (ONT) sequencing. DNA samples prepared as described in the Materials and Methods for the 12 isolates were checked for integrity with gel electrophoresis, using a 0.5 % agarose gel and 1 Kb ladder (Sharp DNA Ladder, Real Biotech Corporation). The quantity of DNA was determined using a Qubit 3.0 Fluorometer (version APPv1.02 and MCUv0.21) and Qubit dsDNA HS Assay kit. Quality of DNA samples was evaluated using a NanoDrop 2000c spectrophotometer (NanoDrop 2000/2000c version 1.6.198). A total of ≥100 ng of DNA per isolate was sent for Illumina WGS at AGRF. The sequencing package was a 1 Gbp Bundle (Prep M) (NGS-DNB-324) per sample with a total sequencing depth of approximately 138×.

Raw FASTQ.gz files were transferred from the AGRF server to a local computer using FileZilla. WGS data was first processed using FastQC (version 0.12.1) to generate reports on quality, abundance, and size of sequenced reads [3,4]. MultiQC (version 1.11) was used to aggregate the FastQC reports [4]. Illumina sequences were trimmed using Trimmomatic (version 0.36.6) and genomes were constructed using SPAdes (version 3.15.5) [3,4]. Contigs were then queried on the pubMLST database for matches and close matches [3,5].

S17. Comparison of Targeted ONT and Illumina WGS

Accuracy of reads was compared between targeted ONT and allele sequences from Illumina WGS similar to the webservice MLST tool on the Center for Genomic Epidemiology, where two sequences would be aligned and the percentage similarity determined using the number of matching aligned base pairs divided by the total number of base pairs. MAAFT (version 7.505) was used to pairwise align corresponding targeted ONT and Illumina MLST DNA sequences [6-8].

S18. Targeted ONT Sequencing ST Determination of B. pseudomallei Isolates Compared to Illumina WGS

Two known ST controls, ST276 and ST814, were included in ONT sequencing, and their expected STs were confirmed (Table S19 and Table S21). Validation using Illumina WGS revealed complete consensus between the two sequencing methods. All MLST sequences for each isolate and overall ST profiles were 100% matched for all 12 isolates between targeted ONT and Illumina WGS (Table S19).

S19. Sequence Similarity between ONT and Illumina Sequences

Table S19 - Table listing sequence similarity between targeted ONT sequencing and extracted alleles from Illumina WGS.

Isolate	ONT ST	Illumina ST	Percentage Similarity of Sequences
27.1.22-15	2070	2070	100%
27.1.22-17	2080	2080	100%
27.1.22-19	2071	2071	100%
4.2.22-9	2072	2072	100%
4.2.22-10	NA*	NA*	100%
22.11.22-2	2073	2073	100%
6.1.23-3	2074	2074	100%
13.1.23-1	2075	2075	100%
1.2.23-4	2076	2076	100%
27.1.22-14	2077	2077	100%
14.1.23-2	2078	2078	100%
16.1.23-1	2079	2079	100%

*Not applicable (NA) as ST cannot be assigned due to incomplete profile from novel *lepA* allele.

S20. PubMLST Sequence Submission

Novel allele sequences were aligned to example pubMLST database allele sequences using MAAFT (version 7.505) and trimmed to correct length as described on PubMLST (https://pubmlst.org/organisms/burkholderia-pseudomallei) for submission [5,6-8]. Novel allele sequences were submitted to the typing pubMLST MLST database for assessment and curation (Table S22). Once assessed by the database curator, novel ST profiles were submitted to the typing pubMLST MLST database for assessment and curation. All complete *B. pseudomallei* ST profiles were submitted to the isolate pubMLST MLST database for curation. STs and PubMLST ID numbers have been listed in Table S21 for each sequenced Goondaloo Creek *B. pseudomallei* isolate.

S21. Creek Isolate IDs, STs, and PubMLST IDs

Table S21 - Goondaloo Creek sequenced isolates and their corresponding ONT ST and PubMLST ID number.

Isolate ID	PubMLST ID	ST	Isolate ID	PubMLST ID	ST
27.1.22-11	6745	283	6.1.23-1	6765	283
27.1.22-12	6746	283	6.1.23-3	6766	2074
27.1.22-13	6747	1966	6.1.23-4	6767	2072
27.1.22-14	6784	2077	6.1.23-5	6768	2071
27.1.22-15	6748	2070	6.1.23-14	6769	2072
27.1.22-16	6749	283	6.1.23-16	6786	283
27.1.22-17	6750	2080	13.1.23-1	6770	2075
27.1.22-18	6751	1966	13.1.23-2	6771	2073
27.1.22-19	6752	2071	13.1.23-3	6772	2072
27.1.22-21	6789	1966	14.1.23-1	6773	2072
27.1.22-22	6785	1966	14.1.23-2	6787	2078
4.2.22-9	6753	2072	15.1.23-1	NA*	Unknown
4.2.22-10	NA*	Unknown	15.1.23-2	6774	283
4.2.22-11	6754	283	15.1.23-3	6775	283
5.7.22-1	6755	276	15.1.23-4	6776	283
27.7.22-1	NA*	Unknown	16.1.23-1	6788	2079
22.11.22-2	6756	2073	16.1.23-2	6777	1664
23.11.22-1	6757	283	18.1.23-1	6778	283
28.11.22-1	6758	1969	1.2.23-1	6779	276
28.11.22-2	6759	283	1.2.23-2	6780	283
28.11.22-4	6760	2080	1.2.23-3	6781	276
28.11.22-5	6761	283	1.2.23-4	6782	2076
1.12.22-1	6762	624	1.2.23-5	6783	283
1.12.22-3	6763	1966	TSV1 (ST276 control)	2270	276
1.12.22-5	6764	2070	TSV2 (ST814 control)	2271	814

*Not applicable (NA) as these isolates have not been submitted due to incomplete profile.

S22. DNA Sequences for lipA and lepA Alleles

Table S22 – DNA sequences for *lipA* and *lepA* alleles determined from ONT sequencing of Goondaloo Creek *B. pseudomallei* isolates.

**Alleles**	**PubMLST Allele Number**	**Sequence Length (bp)**	**DNA Sequence**
lipA	189	402	AAGTGCACGCGCCGCTGCCCGTTCTGCGACGTCGGC<br>CACGGCCGGCCCGATCCGCTCGACGCAGACGAGCCG<br>AAGAACCTCGCGCGCACGATCGCGGCGCTCAAGCTC<br>AAGTACGTGGTGATCACGAGCGTCGACCGCGACGAT<br>CTGCGCGACGGCGGCGCCGGCCACTTCGTCAAGTGC<br>ATCCGCGAAGTGCGCGAGCAGTCGCCCGCGACGCGC<br>ATCGAGATCCTGACGCCGGACTTCCGTGGCCGCCTC<br>GACCGTGCGCTCGCGATCCTGAACGCGGCGCCGCCC<br>GACGTGATGAACCACAATCTCGAAACGGTGCCGCGC<br>CTGTACAAGGAGGCGCGCCCCGGCTCGGACTATGCG<br>CATTCGCTGAAGCTCCTGAAGGATTTCAAGGCGCTG<br>CATCCG
lepA	NA*	489	CACATCGACCACGGCAAGTCGACGCTCGCGGATCGC<br>ATCATCCAGCTTTGCGGCGGCCTGTCCGACCGGGAG<br>ATGGAATCGCAGGTGCTCGACTCGATGGACCTCGAG<br>CGTGAGCGCGGCATCACGATCAAGGCGCAGACCGCC<br>GCGCTCACCTATCGCGCGCGCGACGGCAAGGTCTAC<br>AACCTGAATCTCATCGATACCCCGGGGCACGTCGAT<br>TTCTCGTACGAAGTGAGCCGCTCGCTGTCCGCGTGCG<br>AGGGCGCGCTGCTCGTCGTCGACGCAAGCCAGGGCG<br>TCGAGGCGCAGACGGTCGCGAACTGCTATACGGCGA<br>TCGAGCTCGGCGTCGAGGTGGTGCCCGTCCTCAACA<br>AGATCGATCTGCCGGCGGCGGCGAACCCGGAGAACG<br>CGATCGCCGAGATCGAGGACGTGATCGGCATCGACG<br>CGATGGACGCGGTGCGCTGCAGCGCGAAGACGGGCC<br>TCGGCGTCGAGGACGTGCTC

*Not accepted by PubMLST due to three base pair insertion.

S23. Number and Percentage Totals for Sequenced Creek Isolates

Table S23 – Number and percentage totals of sequenced Goondaloo Creek *B. pseudomallei* isolates for each ST.

**ST**	**Number of Total Sequenced Isolates**	**Percentage of Total Sequenced Isolates (%)**
276	3	6.25
283	15	31.25
624	1	2.083
1664	1	2.083
1966	5	10.417
1969	1	2.083
2070	2	4.167
2071	2	4.167
2072	5	10.417
2073	2	4.167
2074	1	2.083
2075	1	2.083
2076	1	2.083
2077	1	2.083
2078	1	2.083
2079	1	2.083
2080	2	4.167
Unknown	3	6.25

References

1. Baker, A.; Tahani, D.; Gardiner, C.; Bristow, K.L.; Greenhill, A.R.; Warner, J. Groundwater seeps facilitate exposure to *Burkholderia pseudomallei*. *Appl. Environ. Microbiol.* **2011**, *77*, 7243–7246. https://doi.org/10.1128/AEM.05048-11.
2. Baker, A.L.; Warner, J.M. *Burkholderia pseudomallei* is frequently detected in groundwater that discharges to major watercourses in northern Australia. *Folia Microbiol.* **2016**, *61*, 301–305. https://doi.org/10.1007/s12223-015-0438-3.
3. Rachlin, A.; Luangraj, M.; Kaestli, M.; Rattanavong, S.; Phoumin, P.; Webb, J.R.; Mayo, M.; Currie, B.; Dance, D.A.B. Using land runoff to survey the distribution and genetic diversity of *Burkholderia pseudomallei* strains in Vientiane, Laos. *Appl. Environ. Microbiol.* **2021**, *87*, 4. https://doi.org/10.1128/AEM.02112-20.
4. Smith, S.; Marquardt, T.; Jennison, A.V.; D'Addona, A.; Stewart, J.; Yarwood, T.; Ho, J.; Binotto, E.; Harris, J.; Fahmy, M.; et al. Clinical manifestations and genomic evaluation of melioidosis outbreak among children after sporting event, Australia. *Emerg. Infect. Dis.* **2023**, *29*, 2218–2228. https://doi.org/10.3201/eid2911.230951.
5. Jolley, K.A.; Bray, J.E.; Maiden, M.C.J. Open-access bacterial population genomics: BIGSdb software, the PubMLST.org website and their applications. *Wellcome Open Res.* **2018**, *3*, 124. https://doi.org/10.12688/wellcomeopenres.14826.1.
6. Katoh, K.; Standley, D.M. MAFFT multiple sequence alignment software version 7: Improvements in performance and usability. *Mol. Biol. Evol.* **2013**, *30*, 772–780. https://doi.org/10.1093/molbev/mst010.
7. Katoh, K.; Misawa, K.; Kuma, K.-i.; Miyata, T. MAFFT: A novel method for rapid multiple sequence alignment based on fast Fourier transform. *Nucleic Acids Res.* **2002**, *30*, 3059–3066. https://doi.org/10.1093/nar/gkf436.
8. Katoh, K.; Kuma, K.-i.; Toh, H.; Miyata, T. MAFFT version 5: Improvement in accuracy of multiple sequence alignment. *Nucleic Acids Res.* **2005**, *33*, 511–518. https://doi.org/10.1093/nar/gki198.
